# Glucagon-Like Peptide-1 Cleavage Product Improves Cognitive Function in a Mouse Model of Down Syndrome

**DOI:** 10.1523/ENEURO.0031-19.2019

**Published:** 2019-05-09

**Authors:** Stephen M. Day, Wenzhong Yang, Xin Wang, Jennifer E. Stern, Xueyan Zhou, Shannon L. Macauley, Tao Ma

**Affiliations:** 1 Departments of Internal Medicine and Gerontology and Geriatric Medicine, Wake Forest School of Medicine, Winston-Salem, North Carolina 27157; 2 Department of Integrative Physiology & Pharmacology, Wake Forest School of Medicine, Winston-Salem, North Carolina 27157; 3 Department of Neurobiology and Anatomy, Wake Forest School of Medicine, Winston-Salem, North Carolina 27157

**Keywords:** Alzheimer’s disease, Down syndrome, GLP-1 (9-36), LTP, oxidative stress, synaptic plasticity

## Abstract

Currently there is no effective therapy available for cognitive impairments in Down syndrome (DS), one of the most prevalent forms of intellectual disability in humans associated with the chromosomes 21 trisomy. Glucagon-like peptide-1 (GLP-1) is an incretin hormone that maintains glucose homeostasis by stimulating insulin secretion. Its natural cleavage product GLP-1 (9-36) lacks insulinotropic effects and has a low binding affinity for GLP-1 receptors; thus, GLP-1 (9-36) has historically been identified as an inactive metabolite. Conversely, recent work has demonstrated interesting physiological properties of GLP-1 (9-36) such as cardioprotection and neuroprotection. We have previously shown that GLP-1 (9-36) administration enhances neuronal plasticity in young WT mice and ameliorates cognitive deficits in a mouse model of Alzheimer’s disease. Here, we report that systemic administration of GLP-1 (9-36) in Ts65Dn DS model mice of either sex resulted in decreased mitochondrial oxidative stress in hippocampus and improved dendritic spine morphology, increase of mature spines and reduction of immature spines. Importantly, these molecular alterations translated into functional changes in that long-term potentiation failure and cognitive impairments in TsDn65 DS model mice were rescued with GLP-1 (9-36) treatment. We also show that chronic GLP-1 (9-36) treatment did not alter glucose tolerance in either WT or DS model mice. Our findings suggest that GLP-1 (9-36) treatment may have therapeutic potential for DS and other neurodegenerative diseases associated with increased neuronal oxidative stress.

## Significance Statement

Presently no effective treatment exists for cognitive defects in Down syndrome (DS), one of the most common forms of intellectual disability. Here, we studied the effect of the glucagon-like peptide-1 (GLP-1) cleavage product, GLP-1 (9-36), on an established mouse model of DS. We found that GLP-1 (9-36) treatment significantly improved DS-associated memory deficits and synaptic plasticity impairments. Consistently, GLP-1 (9-36) treatment in DS model mice led to decreased levels of mitochondrial reactive oxygen species and improved dendritic spine morphology. Our findings indicate that GLP-1 (9-36) may have therapeutic potential to improve memory and cognition in DS and other neurodegenerative diseases associated with increased neuronal oxidative stress.

## Introduction

Down syndrome (DS) is one of the most common forms of intellectual disabilities with an apparent genetic cause: the trisomic repeat of chromosome 21 (HSA21; Patterson and Costa, 2005; Mégarbané et al., 2009; Lott and Dierssen, 2010 ). Impaired cognition is a hallmark of DS, and there are currently no effective treatments that improve cognitive function in individuals with DS (Patterson, 2009; Lana-Elola et al., 2011; Lott, 2012). Of note, nearly all DS patients develop typical Alzheimer’s disease (AD) neuropathology (i.e., senile plaques and neurofibrillary tangles) by age 40 (Nelson et al., 2011; Head et al., 2016), and many go on to develop age-dependent AD type dementia syndromes later in life (Dierssen, 2012; Zigman, 2013; Bayen et al., 2018).

Increased reactive oxygen species (ROS) is a common feature of many neurologic diseases of cognitive impairment, including DS (Lin and Beal, 2006; Pagano and Castello, 2012; Wang et al., 2014; Guo et al., 2017). Excessive neuronal ROS disrupts molecular and cellular mechanisms underlying memory and synaptic plasticity maintenance and can lead to cognitive impairment (Massaad and Klann, 2011; Guo et al., 2017). Previous studies have demonstrated that decreasing mitochondrial ROS improves memory and hippocampal synaptic plasticity impairments associated with neurodegenerative diseases such as AD (Dumont et al., 2009; Ma et al., 2011; Massaad and Klann, 2011). Therefore, targeting mitochondrial ROS production or clearance may be a feasible strategy to improve cognitive function in individuals with DS.

Glucagon-like peptide-1 (GLP-1) is an incretin hormone released from the L-cells of the distal ileum in response nutrient ingestion. GLP-1 is released into circulation as the “bioactive” GLP-1 (7-36) and has a strong binding affinity to the GLP-1 receptor (GLP1R; Drucker, 2001; Baggio and Drucker, 2007); pancreatic activation of the GLP1R initiates glucose-dependent insulin secretion. Circulating GLP-1 (7-36) is rapidly degraded (half-life <2 min) into GLP-1 (9-36) by dipeptidyl peptidase-4 (Deacon et al., 1995; Hansen et al., 1999; Nikolaidis et al., 2005). GLP-1 (9-36) has a low binding affinity to the GLP1R and exerts no insulinotropic effects (Knudsen and Pridal, 1996; Kuc et al., 2014). Thus, GLP-1 (9-36) had been traditionally considered as an “inactive” waste product of GLP-1 (7-36). However, a growing body of evidence indicates that that GLP-1 (9-36) has essential biological functions separate from its precursor. For example, in human aortic endothelial cells GLP-1 (9-36), not GLP-1 (7-36), reversed high glucose and high free fatty acid-induced ROS production (Giacco et al., 2015). GLP-1 (9-36) also protected *Glp1r-/-* cardiomyocytes from H_2_O_2_-induced cell death while GLP-1 (7-36) did not (Ban et al., 2010). These data suggest that GLP-1 (9-36) may operate via a mechanism distinct from GLP-1 (7-36). Furthermore, we have shown that GLP-1 (9-36) treatment decreased high levels of hippocampal ROS and rescued memory deficits in a mouse model of AD (Ma et al., 2012). Recently, we demonstrated that chronic GLP-1 (9-36) treatment enhances hippocampal long-term potentiation (LTP) in young WT mice (Day et al., 2017).

In the current study, we aim to investigate whether GLP-1 (9-36) treatment can improve DS pathophysiology by administrating GLP-1 (9-36) systemically to a mouse model of DS. A series of experiments were conducted to test effects and relevant cellular/molecular mechanisms of GLP-1 (9-36) treatment on cognitive impairments and synaptic plasticity deficiency in Ts65Dn DS model mice. Our findings suggest that GLP-1 (9-36) may be a novel therapeutic agent in treating DS-associated cognitive dysfunction and synaptic failure.

## Materials and Methods

### Animals

All mice were housed in the Transgenic Mouse Facility at Wake Forest School of Medicine Animal Facility. Mice were kept in compliance with the National Institute of Health (NIH) *Guide for Care and Use of Laboratory Animals*. The facility kept a 12 h light/dark cycle with regular feeding, cage cleaning, and 24 h access to water. Breeder mice were purchased from The Jackson Laboratory. The mouse colony was maintained by breeding Ts65Dn trisomic females (005252) with B6EiC3Sn.BliAF1 males (003647). Ts65Dn mice used in behavioral experiments did not carry the phosphodiesterase 6b (*Pde6b*) gene mutation associated with retinal degeneration. Genotyping was determined by PCR. All experiments were conducted on male and female 9-month-old mice; Ts65Dn mice reliably exhibit cognitive impairments at this age based on our observation and previous report (Faizi et al., 2011).

### GLP-1 (9-36) treatment

GLP-1(9–36) peptide (Eurogentec) was administered daily via intraperitoneal injection at a dose of 500 ng/g/d (Ma et al., 2012). Mice were treated with GLP-1 (9-36) or a saline control for 14 d before behavioral tests began. GLP-1 (9-36) or saline treatment continued throughout behavioral experiments. Mice were then sacrificed for MitoSOX assay, Golgi-Cox staining, Western blot, or electrophysiological experiments. Mice were weighed weekly to determine appropriate drug dose. GLP-1 (9-36) was prepared as stock solution and was diluted to the final concentrations before use.

### Detection of mitochondrial superoxide in hippocampal slices

After completion of either drug or vehicle treatment, mice were killed and 400 μm hippocampal slices were incubated with 5 μm MitoSOX Red, a mitochondrial superoxide indicator (prepared as 5 mm stock solution immediately before the experiments; Invitrogen) for 10 min. Slices then were fixed with ice-cold 4% paraformaldehyde in PBS overnight at 4°C. Slices were further cut into 40 μm sections and mounted onto pre-subbed slides with VECTASHIELD mounting medium with DAPI (Vector Laboratories). The sections were imaged using a Leica TCS SP5 confocal microscope at 20× magnification. All parameters (pinhole, contrast, gain, offset) were held constant for all sections from the same experiment.

### Western blot

Hippocampal slices were flash-frozen on dry ice and sonicated as previously described (Ma et al., 2012). Samples containing equal amounts of protein lysate were loaded on 4–12% Tris-glycine SDS-PAGE gels for standard gel electrophoresis. Membranes were probed overnight at 4°C using the OXPHOS primary antibody (1:250; Abcam) and GAPDH (1:10,000; Cell Signaling). Densitometric analysis was performed using ImageJ software. Data were normalized to GAPDH.

### Golgi–Cox stain

Brains were processed using the FD Rapid GolgiStain Kit in accordance with the manufacturer’s instructions (FD Neurotechnologies; catalog #PK401). Transverse sections (125 μm) were made using a Leica VT1200S vibratome and mounted unto gelatin-coated slides. Development was performed according to kit instructions. Sections were dehydrated through a graded ethanol series and cleared in xylene. Slides were cover-slipped with Vectamount Permanent Mounting Medium (Vector Laboratories; catalog, #H-5000) and imaged at 100X on a Keyence BZ-X710 microscope. For spine analysis, images were blinded, and spines were manually counted and sorted as previously described (Risher et al., 2014).

### Glucose tolerance test

An intraperitoneal glucose tolerance test (IPGTT) was administered to mice before the treatment regimen began and after daily saline or GLP-1 (9-36) treatment (500 ng/g/d). On the day of the experiment, mice were fasted for 4 h and glucose (2.0 g/kg) was administered intraperitoneally. Blood samples were taken from tail veins and blood glucose was measured (0, 15, 30, 45, 60, 90, 120 min, respectively) using a glucometer (Bound Tree Medical Precision XTRA Glucometer; Fisher).

### Hippocampal slice preparation and electrophysiology

Acute 400 μm transverse hippocampal slices were prepared using a Leica VT1200S vibratome as described previously. Slices were maintained at room temperature for at least 2 h in an artificial CSF containing the following (in mm): 118 NaCl, 3.5 KCl, 2.5 CaCl_2_, 1.3 MgSO_4_,1.25NaH_2_PO_4_, and 15 glucose, bubbled with 95% O_2_/5% CO_2_. For electrophysiology, monophasic constant-current stimuli (100 μs) were delivered with a bipolar silver electrode placed in the stratum radiatum of area CA3. Field EPSPs (fEPSPs) were recorded using a glass microelectrode from the stratum radiatum of area CA1. LTP was induced using a high-frequency stimulation (HFS) protocol consisting of two 1 s, 100 Hz trains separated by 60 s. Paired-pulse facilitation (PPF) was conducted using a pair of stimuli with interstimulus intervals (ISIs) of 25, 50, 100, 200, or 300 ms delivered to the CA3, and the respective slopes of the fEPSP were measured. The ratio of the second slope to the first slope, compared with the ISI was plotted. For input/output (I/O) curves, the slopes of fEPSPs and the values of the fiber volley at different stimulation intensities were measured.

### Object location memory task

**Figure 1.**
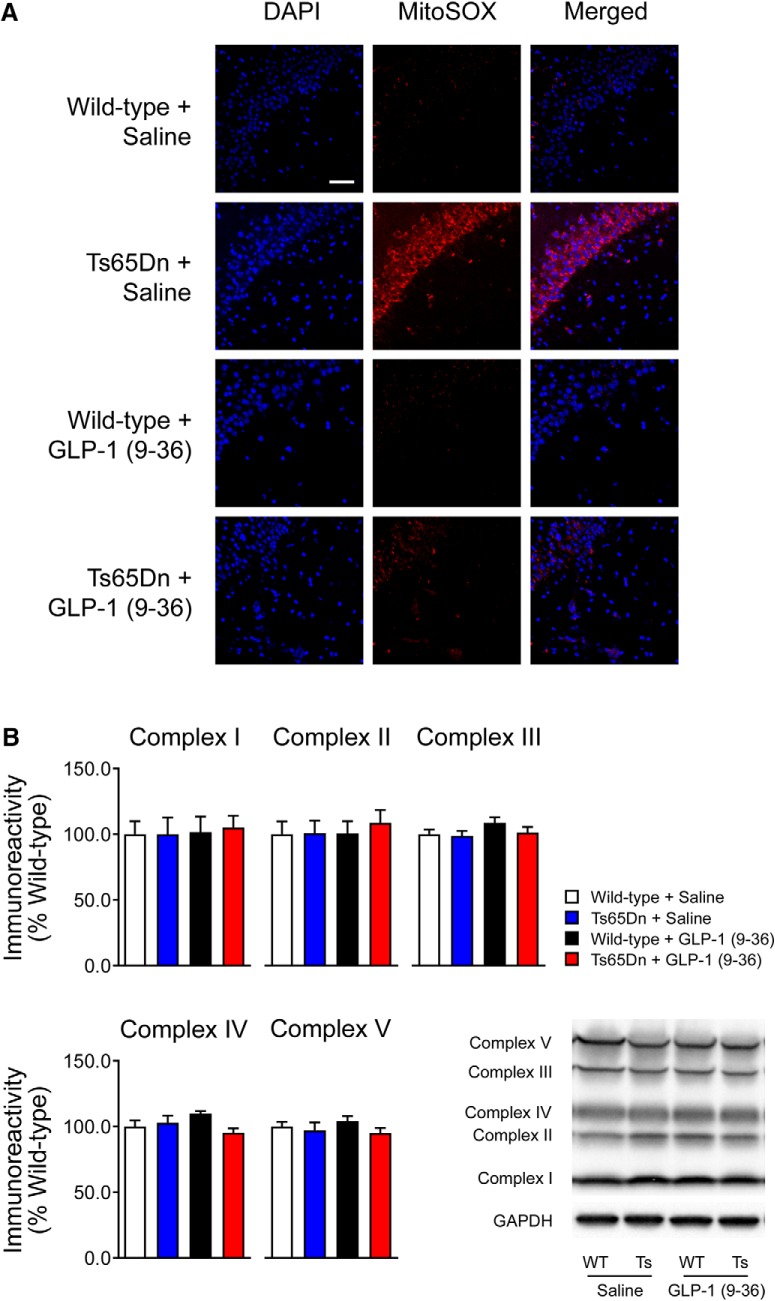
GLP-1 (9-36) treatment normalizes increased hippocampal mitochondrial superoxide in Ts65Dn mice. ***A***, The MitoSOX fluorescent signal (red) was increased in area CA1 of hippocampal slices from Ts65Dn compared with slices from WT mice. GLP-1 (9-36) treatment normalized the DS-associated increased fluorescent signal. Results are representative of three independent experiments (20×, scale bar, 50 μm; *n* = 3 per group). ***B***, Western blot experiment revealed no significant difference in the expression of oxidative phosphorylation complex proteins among all four experimental groups (*n* = 8 per group).

Mice were habituated to an opaque plastic chamber (30 × 21 × 15 cm) with visible spatial cues for 10 min. After 24 h, mice were returned to the chamber with two identical objects in the arena and were allowed to freely explore and interact with the objects for 10 min. Twenty-four hours later, mice were returned to the chamber again, where one of the two objects had been relocated to an adjacent position (Fig. 1*A*). Objects and changes in object location were randomly determined and counterbalanced. Time spent with each object was measured and calculated as a percentage of the total object interaction time. Novel object preference of <50% indicates memory impairments. Time with objects was measured both manually and with EthoVision XT tracking software. Mice with a total object interaction time of <10 s were excluded from analysis. Data collection and analysis were performed blinded.

### Passive avoidance task

For the training phase of the passive avoidance task (PAR apparatus, Panlab), mice were placed in a well-lit chamber (25 × 25 × 24 cm). Following a 60 s exploration period a trap door opened to reveal a smaller, dark chamber (19 × 10 × 12 cm). On entry into the dark chamber the trap door closed, and mice were given a mild foot shock (foot shock intensity: 0.1 mA, 2 s duration). Memory was tested 24 h after training when mice were returned to the light chamber, and following a 60 s exploration period, the trap door opened to reveal the dark chamber. On entry into the dark chamber the trap door closed (no foot shock was given on the test day). For training and test day, mice were given a max latency of 600 s. Mice that did not enter the dark chamber on the first day were not included in data analysis.

### Data analysis

Data were presented as mean ± SEM. Summary data are presented as group means with SE bars. Normality of the data were tested using GraphPad Prism (GraphPad Software), and the criteria for parametric testing were met. For comparisons between two groups, a two-tailed independent Student’s *t* test was performed using GraphPad Prism 7 software. Two-tailed unpaired *t* tests were performed for within-group analyses. For comparisons between more than two groups, two-way repeated measures ANOVA was used with Tukey’s *post hoc* tests for multiple comparisons. Error probabilities of *p* < 0.05 were considered statistically significant.

### Experimental design and statistical analysis

All statistical comparisons were done in four mouse groups: WT+ saline, Ts65Dn + saline, WT+ GLP-1 (9-36), and Ts65Dn + GLP-1 (9-36). The number of mice (*n*) for each comparison is given in the corresponding figure legends. For each tested parameter, data distributions are represented by mean ± SEM. For all tests, results were considered statistically significant at *p* < 0.05. All data analyses and statistics were performed in GraphPad Prism (GraphPad Software).

### Study approval

All methods involving animals were approved by the IACUC of Wake Forest University School of Medicine.

## Results

### GLP-1 (9-36) treatment decreases DS-associated elevated levels of mitochondrial superoxide

Increased neuronal mitochondria-derived ROS is an important feature of DS and is linked to cognitive impairments (Massaad and Klann, 2011; Zis et al., 2012). Previous studies have demonstrated that GLP-1 (9-36) treatment decreases ROS production both in cultured cells and animal models (Ban et al., 2010; Ma et al., 2012; Giacco et al., 2015). To determine whether GLP-1 (9-36) treatment is able to decrease DS-associated ROS, we assessed the levels of mitochondrial ROS by staining live slices with MitoSOX Red, a fluorogenic dye that selectively detects mitochondria-specific superoxide, a primary ROS (Ma et al., 2012; Wong et al., 2015; Ishii et al., 2017). Compared with WT mice, Ts65Dn mice demonstrated significantly enhanced MitoSOX fluorescence signal, indicating increased levels of mitochondrial superoxide and ROS (Fig. 1*A*). Notably, GLP-1 (9-36) treatment blunted the DS-associated increase in mitochondrial superoxide (Fig. 1*A*). We further examined the effects of GLP-1 (9-36) treatment on mitochondrial electron transport chain (ETC) proteins using Western blot assay. The ETC consists of a series of protein complexes that transfer electrons from electron donors to electron acceptors via redox reactions. The ETC proteins create the proton gradient that drives ATP production; however, ETC complexes I and III produce large amounts of superoxide in this process (Massaad and Klann, 2011). We observed no differences in ETC expression according to either genotype or treatment condition (Fig. 1*B*), indicating that the difference in superoxide production was not because of dysregulations of ETC proteins expression. These findings are also consistent with previous reports demonstrating that GLP-1 (9-36) has antioxidant-like properties (Giacco et al., 2015).

### GLP-1 (9-36) treatment improves dendritic spine morphology in Ts65Dn mice

**Figure 2.**
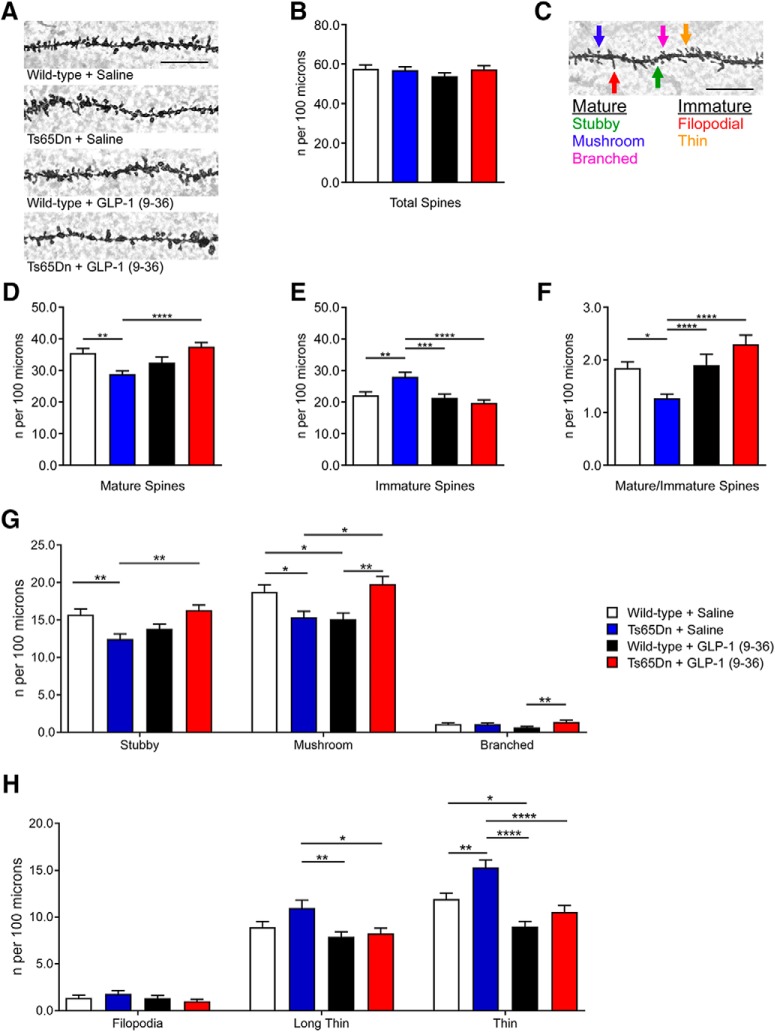
GLP-1 (9-36) treatment improves dendritic spine morphology of hippocampus in Ts65Dn mice. ***A***, Representative images from Golgi-Cox stain of area CA1 dendritic spines (100×, scale bar, 12.5 μm). ***B***, Total area CA1 dendritic spine density (counts per 100 μm) was unaltered among all experimental groups. ***C***, Diagram depicting dendritic spine classification based on previous publication (100×, scale bar, 12.5 μm). ***D***, Mature spine density (counts per 100 μm) was decreased in Ts65Dn mice compared with WT group, and was improved with treatment of GLP-1 (9-36). Stubby, mushroom, and branched spine types were classified as mature. ***E***, Immature spine density (counts per 100 μm) was increased in Ts65Dn mice compared with WT group, and was restored with GLP-1 (9-36) treatment. Filopodial and thin type spies were classified as immature. ***F***, Mature–immature spine ratio was reduced in Ts65Dn mice compared with WT group, and was restored with GLP-1 (9-36) treatment. ***G***, Subclassification of mature spines. ***H***, Subclassification of immature spines (WT+ Saline: *n* = 81 dendrites; Ts65Dn + Saline; *n* = 80 dendrites; WT+ GLP-1 (9-36): *n* = 51 dendrites: Ts65Dn + GLP-1 (9-36): *n* = 81 dendrites). *p < 0.05, **p < 0.01, ***p < 0.001, ****p < 0.0001; one-way ANOVA with Tukey’s *posthoc* test. Values represent mean ± SEM.

Dendritic spine morphology regulation is vital to synaptic integrity and associated with neural plasticity and memory formation (Hering and Sheng, 2001; Sala and Segal, 2014). Excessive ROS can also affect regulation of spine morphology (Dos Reis et al., 2013). Using the rapid Golgi–Cox staining protocol, we assessed spine density and morphology changes of dendritic spines within the stratum radiatum of hippocampal CA1 area. Although there were no differences in overall dendritic spine density between groups (Fig. 2*A*,*B*), we further analyzed changes in spine morphology based on published guidelines (“mature” vs “immature”; Fig. 2*C*; Risher et al., 2014), and found significant morphologic differences. Compared with WT mice, Ts65Dn mice showed a significantly lower density of mature spines (WT+ saline vs Ts65Dn + saline: *p* = 0.0014) and a significantly higher density of immature spines (WT+ saline vs Ts65Dn + saline: *p* = 0.0013; WT + GLP-1 (9-36) vs Ts65Dn + saline: *p* = 0.0016; Fig. 2*C*–*E*). Markedly, GLP-1 (9-36) treatment restored spine morphology (both mature and immature) in Ts65Dn mice to WT norms (Fig. 2*D*–*E*). These differences in spine morphology became apparent when we calculated the mature/immature spine ratio for each group. We found that saline- and GLP-1 (9-36)-treated WT mice had a significantly higher mature/immature spine ratio than saline-treated Ts65Dn mice (WT+ saline vs Ts65Dn + saline: *p* = 0.0155; WT + GLP-1 (9-36) vs Ts65Dn + saline: *p* = 0.0222; Fig. 2*F*). Importantly, Ts65Dn mice treated with GLP-1(9-36) had a significantly higher mature–immature spine ratio than Ts65Dn mice treated with saline (*p* < 0.0001; Fig. 2*F*). Furthermore, we characterized and quantified each individual category of the spine data (Fig. 2*G*,*H*). Of interest, for branched mature and filopodia immature spines, we did not observe any significant DS-associated alternations or rescuing effects of GLP-1 (9-36) (Fig. 2*G*,*H*). Together, these data indicate beneficial effects of GLP-1 (9-36) treatment on DS-associated impairments of synaptic structure, which may represent a mechanism through which GLP-1 (9-36) improves cognition and synaptic function.

### GLP-1 (9-36) treatment does not affect glucose clearance in Ts65Dn or WT mice

**Figure 3.**
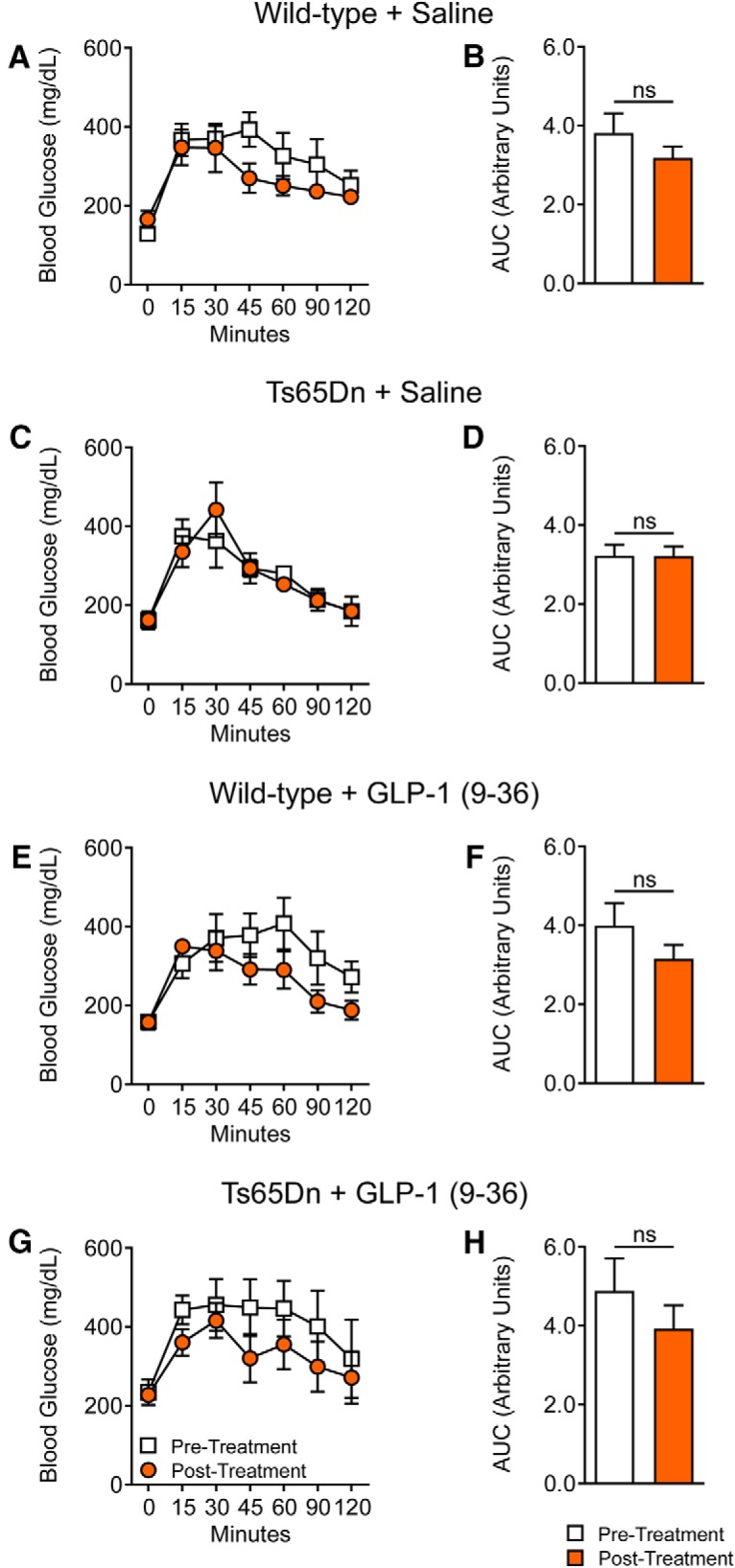
GLP-1 (9-36) treatment does not affect glucose clearance following glucose tolerance tests. Glucose tolerance test (GTT) of WT and Ts65Dn mice was performed before and after saline or GLP-1 (9-36) treatments. ***A***, ***C***, ***E***, ***G***, Two-way ANOVA revealed no significant differences between pretreatment or post-treatment in glucose response curves among all four experimental groups. ***B***, ***D***, ***F***, ***H***, Independent *t* test revealed no significant differences between pretreatment or posttreatment GTT AUC. WT + Saline: *n* = 6; Ts65Dn + Saline: *n* = 5; WT + GLP-1 (9-36): *n* = 8; Ts65Dn + GLP-1 (9-36): *n* = 6.

GLP-1 (7-36), the precursor peptide of GLP-1(9-36), is known as an insulinotropic hormone; it enhances insulin secretion and promotes glucose clearance in humans. In contrast, GLP-1 (9-36) has low binding affinity for the GLP-1R and usually is not considered to have any insulinotropic effects (Vahl et al., 2003). Nevertheless, given that we are treating mice with GLP-1 (9-36) at supraphysiological doses, we sought to determine whether GLP-1 (9-36) affects glucose tolerance at the dose administered. To measure this, we performed an IPGTT (Macklin et al., 2017). As shown in Figure 3, saline or GLP-1 (9-36) treatment did not alter glucose tolerance in both WT and Ts65Dn mice, as indicated by measurement of the glucose x time clearance curve and the area under the curve (AUC; Fig. 3*A*–*H*).

### GLP-1 (9-36) treatment rescues hippocampal synaptic plasticity impairments in Ts65Dn mice

**Figure 4.**
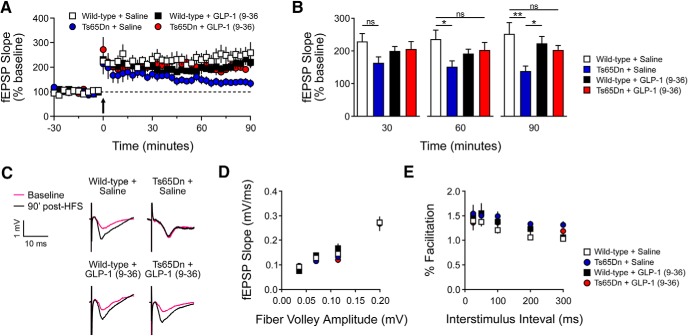
GLP-1 (9-36) treatment rescues hippocampal synaptic plasticity impairments in Ts65Dn mice. ***A***, Acute hippocampal slices from WT+ Saline (*n* = 9), Ts65Dn + Saline (*n* = 10), WT+ GLP-1 (9-36) (*n* = 10), and Ts65Dn + GLP-1 (9-36) (*n* = 9) mice were stimulated with HFS to induce LTP at the CA3-CA1 synapse. Arrow indicates HFS. Two-way repeated-measures ANOVA revealed significant group (*p* < 0.05), time (*p* < 0.05), and group × time (*p* < 0.001) effects. At 90 min post-HFS, Tukey’s *post hoc* tests revealed Ts65Dn + Saline had significantly impaired LTP compared with GLP-1 (9-36)-treated Ts65Dn mice (p < 0.05) and saline- and GLP-1 (9-36)-treated WT mice (*p* < 0.001, *p* < 0.05). ***B***, Measurement of fEPSP slope at 30, 60, and 90 min after HFS (*p < 0.05, **p < 0.01). ***C***, Representative fEPSP traces at baseline and 90 min post-HFS. ***D***, I/O curves were established by plotting fEPSP amplitudes against fiber volley amplitudes at increasing stimulus intensities in hippocampal slices from WT+ Saline (*n* = 9), Ts65Dn + Saline (*n* = 10), WT+ GLP-1 (9-36) (*n* = 9), and Ts65Dn + GLP-1 (9-36) (*n* = 9) mice. One-way ANOVA revealed no significant differences between groups at any time point. ***E***, PPF in WT+ Saline (*n* = 9), Ts65Dn + Saline (*n* = 10), WT+ GLP-1 (9-36) (*n* = 9), and Ts65Dn + GLP-1 (9-36) (*n* = 9) mice. One-way ANOVA revealed no significant differences between groups at any time point.

Previous studies revealed hippocampal synaptic plasticity impairments in Ts65Dn mice (Kleschevnikov et al., 2004; Costa and Scott-McKean, 2013). We next performed electrophysiological experiments to determine whether GLP-1 (9-36) treatment could improve DS-associated deficits of LTP, an established form of synaptic plasticity and a cellular model for learning and memory (Klann and Dever, 2004; Kandel et al., 2014). Although WT mice from both treatment groups [saline and GLP-1(9-36)] showed robust, sustained LTP induced by HFS, saline-treated Ts65Dn mice exhibited declined LTP (Fig. 4 *A*–*C*). Of note, LTP impairments in DS mice were significantly rescued by treatment with GLP-1 (9-36) (Fig. 4 *A*–*C*). GLP-1 (9-36) “appears” to have depressing but nevertheless insignificant effects on LTP of WT slices (Fig. 4*A*,*B*). In addition, we assessed the effects of GLP-1 (9-36) treatment on basal synaptic transmission by inducing synaptic responses with a range of stimulus intensities, and observed similar synaptic I/O relationship among all groups (Fig. 4*D*). We also investigated PPF, a form of calcium-dependent presynaptic plasticity evoked by two temporally-linked stimuli at various intervals (Katz and Miledi, 1968), and did not find any differences in PPF among all four experimental groups (Fig. 4*E*). These findings indicate that GLP-1 (9-36) can improve DS-associated synaptic plasticity impairments without affecting basal synaptic transmission.

### GLP-1 (9-36) treatment alleviates cognitive deficits in Ts65Dn mice

**Figure 5.**
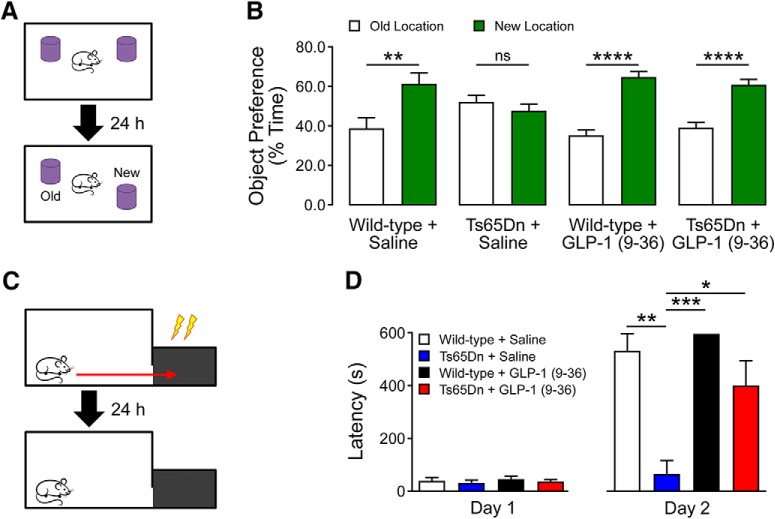
GLP-1 (9-36) treatment alleviates cognitive deficits in Ts65Dn mice. ***A***, Schematic of OLM task and object preference for familiar and new locations during the test session. ***B***, Spatial memory was impaired in Ts65Dn mice compared with WT controls, and the impairments were rescued with GLP-1 (9-36) treatment. Preference for the new location <50% of the total interaction time indicates cognitive impairment (WT+ Saline: *n* = 10; Ts65Dn + Saline: *n* = 11; WT+ GLP-1 (9-36): *n* = 12; Ts65Dn + GLP-1 (9-36): *n* = 13; **p < 0.01, ***p < 0.001, independent *t* test). ***C***, Schematic of passive avoidance paradigm and latency to dark compartment on Day 1 and Day 2 of the test. ***D,*** No differences were observed in latency to dark compartment on acquisition day (Day 1; one-way ANOVA; *p* > 0.05). On test day (Day 2), Ts65Dn mice had a significantly shorter latency to enter into the dark compartment than WT control groups, indicating cognitive impairments. In contrast, GLP-1 (9-36) treatment significantly increased latency to the dark compartment in Ts65Dn mice, indicating improved cognitive function. None of the WT mice treated with GLP-1 (9-36) entered the dark compartment on testing day. (WT+ Saline: *n* = 7; Ts65Dn + Saline: *n* = 3; WT+ GLP-1 (9-36): *n* = 9; and Ts65Dn + GLP-1 (9-36): *n* = 7). One-way repeated-measures ANOVA with Tukey’s *post hoc* tests; *p < 0.05, **p < 0.01, ***p < 0.001, ****p < 0.0001.

To determine whether GLP-1 (9-36) treatment can rescue DS-associated cognitive deficits, we treated Ts65Dn mice and their WT littermates with GLP-1 (9-36) (500 ng/g/d; i.p.) or a saline control continuously for 14 d (Day et al., 2017). We then subjected mice to a series of behavioral tasks to evaluate their cognitive performance. We assessed the spatial learning and memory by testing the mice on object location memory (OLM) task (Kleschevnikov et al., 2012). In the OLM test, WT mice treated with either saline or GLP-1 (9-36) exhibited normal cognition, spending a significantly greater percentage of time interacting with the relocated object than with the object in the old location (Fig. 5*A*,*B*). In contrast, Ts65Dn mice treated with saline failed to recognize the relocated object and spent similar amounts of time with either object, indicating impaired spatial learning and memory. Notably, Ts65Dn mice treated with GLP-1 (9-36) displayed normal cognition, as indicated by a preference for objects in the novel location (Fig. 5*B*).

Moreover, we assessed whether GLP-1 (9-36) treatment would affect long-term associative memory using a 2 d passive avoidance paradigm (Shi and Sun, 2012; Fig. 5*C*). During the acquisition phase (Day 1), mice received a mild foot shock (0.2 mA for 1 s) on entering the dark chamber; mice from all groups exhibited similar latency to enter the dark chamber on day 1 (Fig. 5*D*). On the test day (Day 2), WT mice treated with saline or GLP-1 (9-36) were averse to enter the dark chamber (long latency), indicating normal cognition. Compared with WT mice, the latency to enter the dark chamber was much shorter in saline-treated Ts65Dn mice, indicating cognitive impairment (WT+saline vs Ts65Dn+saline: *p* = 0.0033; WT+GLP-1 (9-36) vs Ts65Dn+saline: *p* = 0.0007; Fig. 5*D*). Importantly, Ts65Dn mice treated with GLP-1 (9-36) displayed improved cognition, as indicated by a significantly longer latency entering the dark chamber, compared with saline-treated group (Ts65Dn+saline vs Ts65Dn+GLP-1 (9-36): *p* = 0.0455; Fig. 5*D*). Together, these data indicate that GLP-1 (9-36) treatment can rescue the cognitive deficits in aged, Ts65Dn DS model mice.

## Discussion

In the current study, we have shown that GLP-1 (9-36), a natural peptide and the primary cleavage product of the incretin hormone GLP-1 (7-36), ameliorates cognitive deficits and synaptic plasticity impairments in a mouse model of DS. With regards to potential molecular/cellular mechanisms, we found that GLP-1 (9-36) treatment decreased the DS-associated elevated mitochondrial superoxide, a major ROS that is linked to memory impairments and synaptic failure (Massaad and Klann, 2011; Guo et al., 2017). We also observed that GLP-1 (9-36) treatment led to increased mature dendritc spines in hippocampus. Together, these findings demonstrate that GLP-1 (9-36) may exert beneficial effects on the molecular mechanisms underlying impairments of memory and synaptic plasticity in DS.

This is the first study we are aware of that has characterized dendritic spine morphology in Ts65Dn mouse model of DS. Spines are dynamic structures whose morphologic structure is directly related to function (Berry and Nedivi, 2017), representing an essential mechanism underlying memory and synaptic plasticity. Immature spines are highly dynamic structures that have little function in synaptic transmission but are essential in initiating contact with nearby axons to form synaptic connections (Lohmann et al., 2005). Furthermore, synaptic weakening induced by long-term depression destabilizes and eliminates existing spines (i.e., increases immature spine density; Nagerl et al., 2004; Zhou et al., 2004). Our findings that DS model mice have increased immature spine density are in line with previous work demonstrating that DS model mice have increased GABAergic innervation to the hippocampus (Mojabi et al., 2016). Conversely, mature spines form stable connections with presynaptic terminals, and are highly sensitive to glutamatergic transmission (Zito et al., 2009; Berry and Nedivi, 2017). Previous studies have shown that excitatory, glutamatergic transmission increases spine volume and stabilizes newly-formed spines (i.e., increases mature spine density; Lang et al., 2004; Kopec et al., 2006; Zito et al., 2009). The spine’s ability to alter their morphology in response to increased or decreased synaptic activity has led some to categorize immature spines as “learning” spines, and mature spines as “memory” spines (Bourne and Harris, 2007). The increased immature spine density in saline-treated Ts65Dn mice may explain their memory impairments. Thus, the shift in spine composition (mature ≫ immature) observed in GLP-1 (9-36)-treated Ts65Dn mice may provide a mechanistic explanation for their improved memory and synaptic plasticity.

That the alterations in dendritic spine morphology were associated with decreased mitochondrial oxidative stress with GLP-1 (9-36) treatment is especially interesting. Increased neuronal oxidative stress is a pathologic hallmark of DS and is associated with aging and age-related neurodegenerative diseases (Zana et al., 2007; Dumont et al., 2009; Ma et al., 2011; Massaad and Klann, 2011; Zis et al., 2012; Wang et al., 2014; Panel et al., 2018). AD model mice have elevated mitochondrial superoxide production and impaired memory and synaptic plasiticity (Ma et al., 2012). The association beetween oxidative stress and dendritic spine morphology remains elusive. One study found that a single dose of cranial irradiation, which causes a persistent increase in ROS, caused significantly decreased immature spines and decreased mature spines (Chakraborti et al., 2012). Another study showed that rats with excessive neuronal ROS had a low mature spine density, which was restored with antioxidant treatment (Dos Reis et al., 2013). In the context of our own work, these studies demonstrate a possible causative relationship between excessive ROS and dendritic spine morphology in DS. Meanwhile, future studies are warranted to determine the detailed molecular mechanisms through which oxidative stress interfers with spine maturation in DS.

Currently, there are no effective pharmacological therapies capable of improving cognition in DS patients. We are excited by the findings that GLP-1 (9-36) treatment is able to correct aberrant cognition and synatpic plasticity in the DS model mice. Moreover, the observation that DS-associated increased levels mitochondrial superoxide is blunted by GLP-1 (9-36) treatment agrees with a body of evidence linking mitochondrial ROS to aging-related cognitive and synatpic pathology (Massaad and Klann, 2011; Wang et al., 2014; Guo et al., 2017). Previous studies have attempted to improve cognitive decline in DS patients with antioxidant treatment. Although preclinical studies have found success, they have yet to prove effective in clinical trials (Ani et al., 2000; Lott et al., 2011; Sano et al., 2016). These clinical trials have attempted to improve cognition through either nutritional supplementation or with traditional ROS scavengers such as α-tocopherol (vitamin E; Ani et al., 2000; Lott et al., 2011; Sano et al., 2016). A potential problem with this approach is that it only prevents ROS from oxidizing cellular components, but does nothing to prevent the excessive ROS genesis. Conversely, as we and others have shown, GLP-1 (9-36) protects against oxidative stress and decreased ROS production. It has also been shown to decrease oxidative stress in animal models of cognitive disease, known to have increased ROS production (Ban et al., 2010; Ma et al., 2012; Giacco et al., 2015). Therefore our findings here, in conjuction with previous studies (Ma et al., 2012), suggest that the natural peptide and GLP-1 cleavage product GLP-1 (9-36) has therapeutic potential to improve cognition in DS and other neuronal diseases associated with excessive mitochondrial ROS.

## Synthesis

Reviewing Editor: Menno Witter, Norwegian University of Science and Technology Decisions are customarily a result of the Reviewing Editor and the peer reviewers coming together and discussing their recommendations until a consensus is reached. When revisions are invited, a fact-based synthesis statement explaining their decision and outlining what is needed to prepare a revision will be listed below. Note: If this manuscript was transferred from JNeurosci and a decision was made to accept the manuscript without peer review, a brief statement to this effect will instead be what is listed below.

This is a transferred manuscript from J. Neuroscience and in view of the comments of the two reviewers, I have taken the decision to review the revised version myself. Although the authors have responded appropriately to several of the comments there are two methodological points that they have essentially evaded. These need to be addressed before the paper can be accepted.

1) this relates to the second comment of reviewer 1: ‘More details about statistics should be provided. For instance, were tests done to determine whether parametric statistics were appropriate for these data sets?

The current version of the manuscript does not provide the relevant information in the method section to assess this issue raised by the reviewer. So this needs to be changed. Referring to another paper of the same group is insufficient. The authors have to report whether they did test if criteria for parametric testing were met. Note by the way that there is no reference in the method section to Ma et al! Simply describe that you tested for normality of the data.

2) This refers to the second comment of reviewer 2: “Fig 2 (all components). The authors should use a quantitative assessment for spine length. Their qualitative (“mature” vs “immature”) is too subjective. While I don't doubt their findings, I think their primary data could be presented better.”

The response of the authors is rather evasive and insufficient; In the method section it is stated: “For spine analysis, images were blinded, and spines were manually counted and sorted as previously described (Risher et al., 2014). The method described by Risher is actually a quantitative measure using software based analyses such that several morphological features are actually quantified. So referring to a paper as an appropriate method, but not using that method, is incorrect. The authors should be open about what they have done, a qualitative observation, based on a morphological description of spine attributes, for which they can of course refer to Risher, but they should describe in the results what criteria were used. In case they have not quantified, so rely only on a qualitative assessment, it is considered common practice to have two independent observers analyze the data and report observer variation.

Finally, with respect to the comments by reviewer 2 about figure 4, The authors are correct in their response, but the fact that the reviewer brings this up, points to the possibility of misinterpretation of the figure. So I suggest to add one sentence to the result section, emphasizing that what seems to be an effect (A) is not significant (B).
